# Oral Health-Related Quality of Life, Dry Mouth Sensation, and Level of Anxiety in Elderly Patients Rehabilitated with New Removable Dentures

**DOI:** 10.1055/s-0041-1735796

**Published:** 2021-11-23

**Authors:** Lisiane Cristina Bannwart, Clóvis Lamartine de Moraes Melo Neto, Marcelo Coelho Goiato, Daniela Micheline dos Santos, Cristina Aparecida da Silva Paiva, Nathaly Vilene de Araújo Moreno, Emily Vivianne Freitas da Silva, André Pinheiro de Magalhães Bertoz

**Affiliations:** 1 Department of Dental Materials and Prosthodontics, São Paulo State University, School of Dentistry, Araçatuba, São Paulo, Brazil; 2 Paulista University, School of Dentistry, Manaus, Amazonas, Brazil; 3 Oral Oncology Center, School of Dentistry, São Paulo State University, Araçatuba, São Paulo, Brazil; 4 Department of Pediatric and Social Dentistry, São Paulo State University, School of Dentistry, Araçatuba, São Paulo, Brazil

**Keywords:** complete denture, removable partial denture, quality of life, satisfaction, xerostomia, anxiety, questionnaires, Geriatric Oral Health Assessment Index, Visual Analog Scale, State-Trait Anxiety Inventory

## Abstract

**Objective** This study aimed to evaluate the influence of new complete dentures (CDs) and new removable partial dentures (RPDs) on oral health-related quality of life, dry mouth sensation, and anxiety level of their wearers.

**Materials and Methods** In total, 20 complete edentulous patients (in both arches) that needed to be rehabilitated with new CDs, and 20 partial edentulous patients (in both arches) that needed to be rehabilitated with new RPDs, were included in this study. Each patient must wear the same pair of CDs or RPDs for more than 5 years. Oral health-related quality of life, dry mouth sensation, and level of anxiety were assessed by using the following questionnaires: Geriatric Oral Health Assessment Index (GOHAI), VAS (Visual Analog Scale) Xerostomia Questionnaire, and State-Trait Anxiety Inventory (STAI). These questionnaires were applied before oral rehabilitation (initially initial time point) and 3 months after insertion of new dentures (end time point).

**Statistical Analysis** For the results of STAI-State, STAI-Trait, and GOHAI, the Wilcoxon test was applied to compare the time points. For the results of the VAS xerostomia questionnaire, two-way repeated measures ANOVA (analysis of variance) was applied, followed by the Tukey test. The *p* -values lower than 0.05 were considered statistically significant.

**Results** / **Conclusion** For both groups, it was observed that 3 months after the insertion of new removable dentures: (1) there was an increase in oral health-related quality of life; (2) there was a reduction in anxiety (trait anxiety and state anxiety); and (3) there was a perception of greater salivation.

## Introduction

In 2010, the world population aged 65 years and over was estimated to be 524 million (∼8% of the total world population in 2010). 1 2 3 4 By 2050, it is estimated that that number will be higher than 1.5 billion, representing approximately 16% of the total global population. 1 2 3 4 This number of elderly will represent approximately 16% of the total global population by 2050. 1 2 3 4 Furthermore, complete edentulism may be related to individuals aged 50 years or older. 1 2 3 4 It is noteworthy that the loss of all teeth is usually a gradual process. Thus, an individual before being a complete edentulous person, he/she went through a phase of partial edentulism. Possibly, edentulism (complete and partial) will still be a worldwide problem in the future.

The loss of teeth can impair the aesthetics, function (e.g., chewing and digestion), and phonation, 5 6 favoring the development of psychological disorders in the individual. 6 In addition, nutritional deficit, hypertension, cognitive impairment, worsening self-esteem, and increased risk of mortality were correlated with tooth loss. 6 Therefore, it can have a negative impact on the individual's quality of life.

After the loss of teeth, the residual bone ridge has a very important role for stability and retention of a removable denture. 7 However, the residual bone ridge undergoes a process of continuous resorption, affecting the stability and retention of the denture. 7 It is worth mentioning that the resorption of the upper bone ridge can be up to four times greater than the lower bone ridge. 7 Thus, complete dentures (CDs) and removable partial dentures (RPDs) are usually replaced every 5 years. 8 9

Xerostomia (dry mouth) is another factor that can negatively influence a patient's quality of life, 10 since saliva is an essential substance in the oropharyngeal environment. 10 11 It lubricates, facilitates the swallowing of food, initiates the digestion process, stimulates the perception of flavors, has antimicrobial properties, cleans teeth, and contributes to the retention of dentures. 10 The presence of saliva also helps to prevent tooth decay, candidiasis, erosion, and ulceration of tissues mucous membranes, dysgeusia, dysphagia, gingivitis, and strong halitosis. 10

Removable dentures (CDs and RPDs) have a low cost compared with implant *-* retained prostheses, and are widely used by complete and partial edentulous patients. 5 In addition, patients who have medical or dental contraindications or who fear surgical procedures related to implant placement can be rehabilitated with removable dentures (CDs and RPDs), justifying clinical studies of these types of prostheses.

A literature search was performed to verify if there were studies evaluating CD wearers (CDs on both arches) and RPD wearers (RPDs on both arches) by using the Geriatric Oral Health Assessment Index (GOHAI), the VAS (Visual Analog Scale) Xerostomia Questionnaire, and/or State-Trait Anxiety Inventory (STAI). No studies were found after by using these keyword combinations: “complete” and “partial” and “denture” and “geriatric oral health assessment index” or “state anxiety inventory” or “trait anxiety inventory” or “state-trait anxiety inventory” or “visual analog scale.” Therefore, the objective of this study was to evaluate the influence of new CDs and new RPDs on oral health-related quality of life, dry mouth sensation, and anxiety level of their wearers.

## Materials and Methods

Sixty-two patients from the Clinic of Dental Prosthesis of the Paulista University (School of Dentistry, Manaus, Amazonas, Brazil) were selected. After applying the inclusion and exclusion criteria, 40 patients were included in this study. Among the 40 selected patients, 20 were completely edentulous in both arches and 20 were partially edentulous in both arches.

### Inclusion Criteria

- Age older than 60 years.
- ASA (American Society of Anesthesiology) I and ASA II (controlled systemic disease) patients. 12
- For CD wearers:
  Patients completely edentulous in both arches.
  New CDs must be fabricated for both arches of each patient.
  Wear the same pair of CDs for more than 5 years.
  The maxillary ridge must provide retention to the upper CD without the need to use a denture fixative adhesive. The bone height of the lower edge was random.
- For RPD wearers:
  Patients partially edentulous in both arches.
  New RPDs must be fabricated for both arches of each patient.
For each patient, each of their dental arches could be class I, II, III, or IV according to the Kennedy classification. 13
  Wear the same pair of RPDs for more than 5 years.
  Abutment teeth could be absent, as long as these missing teeth do not impair the use of RPD.
- The following situations must be observed:
  Old dentures must present wear of the acrylic resin teeth.
  Loss of vertical dimension of occlusion.
  Loss of stability and retention of old dentures.
- Individuals must wear their old dentures to chew food. 14
- Healthy oral tissue to support dentures. 14
- Absence of temporomandibular dysfunction, 14 confirmed by the RDC/TMD questionnaire (Research Diagnostic Criteria for Temporomandibular Disorders). 15
- Cognitive ability to answer questionnaires and understand commands.

### Exclusion Criteria

- Patients with upper RPD and lower CD, or vice versa.
- Use of a denture fixative adhesive.
- Old dentures (RPDs or CDs) that cause lesions, pain, and/or bleeding in the oral mucosa during feeding of their wearers.
- Patients who require porcelain teeth for new dentures.
- Patients with osseointegrated dental implants 14 or who required dental implants.
- History of oral surgery in the last 3 months. 14
- Removable denture with relining material on its surface.
- Patients who could not wear their old removable dentures due to their fracture.
- Presence of head and neck cancer.
- Maxillary and/or mandibular torus.
- Alzheimer's disease. 10
- Parkinson's disease. 10
- Immunocompromised patients (e.g., human immunodeficiency virus infection). 10
- Patients who were undergoing radiotherapy or/and chemotherapy. 10 11 16 17
- Abuse of alcohol consumption.
- Use of illicit drugs.
- For RPD wearers:
  Tooth mobility and/or periapical lesion.
  Presence of remaining dental roots.
  Need for dental restorations.
- Patients with a history of psychiatric disorders (e.g., depression), 6 neurological disease, salivary gland disease, and Sjogren's syndrome. 10
- Use of psychiatric drugs that cause xerostomia. 10 11
- Allergy to polymethyl methacrylate.
- Smokers. 18
- Individuals with partial or total dependency of care by third parties.

### Manufacturing of Dentures

All new dentures (CDs and RPDs) were performed by the same operator (specialist in the field of dental prosthesis). New CDs were performed by following the procedures recommended by Zarb et al, 19 while new RPDs were performed by following the procedures recommended by Carr and Brown. 20

All new dentures were manufactured with acrylic teeth (Trilux, VIPI, Brazil) and thermally polymerized resin (Clássico, Brazil) by using the conventional method (hot water baths). 14

### Adjustment and Cleaning of Dentures

After insertion of the new dentures, patients were instructed not to use any type of denture fixative adhesive until the end of the research. This was important so that the patient's perception of their new oral condition was not influenced.

All patients received guidance on the use and cleaning of their dentures. 19 20 Denture adjustments 19 20 21 were performed initially every 2 days and subsequently every 4 days.

### Questionnaires

The questionnaires were applied before oral rehabilitation (initial time point) and 3 months after insertion of new dentures (end time point).

**Oral Health-Related Quality of Life**

The GOHAI was developed by Atchison and Dolan 22 and validated for the Portuguese language (Brazil). 23 This index was developed specifically for the elderly population, and assessed oral health-related quality of life based on the last 3 months experienced by the patient. 22 24 The GOHAI is composed of 12 questions, which are divided into three domains: (1) physical function (feeding, speech, and swallowing); (2) pain or discomfort; and (3) psychosocial function (appearance and social relationship). 23 For each GOHAI question, one of the following answers can be given: “never,” “sometimes,” or “always.” 24 For the statistical evaluation, each answer represented a score: (1) “never,” (2) “sometimes,” and (3) “always.”

**Level of Anxiety**

The STAI was developed by Spielberger et al 25 and validated for the Portuguese language (Brazil). 26 The STAI scale consists of 40 statements about the participant's feelings, which divided into two parts 25 :

- Part I (STAI-State): This part consists of 20 statements and assesses how the individual feels “right now” or “at this moment.” 25 Subjects respond to each item by rating themselves on a 4-point scale. 25 The four categories for the STAI-State scale are (1) not at all, (2) somewhat, (3) moderately so, and (4) very much so. 25
- Part II (STAI-Trait): This part consists of 20 statements and assesses how the individual generally feels. 25 Subjects respond to each item by rating themselves on a 4-point scale. 25 The four categories for the STAI-Trait scale are (1) almost never, (2) sometimes, (3) often, and (4) almost always. 25

For the statistical evaluation, each answer represented a score as shown previously.

**Dry Mouth Sensation**

The VAS that assesses xerostomia was developed by Pai et al. 11 The VAS xerostomia questionnaire subjectively evaluates two main aspects: (1) dryness of oral mucosa (lips, mouth, tongue, or throat), and (2) oral functional ability caused by dryness (difficulty in swallowing and speaking). 11 Patients were instructed to answer each item by marking a vertical line on a 50-mm horizontal scale.

Eight-item VAS xerostomia questionnaire 11 :

**Table:** 

1. Rate the difficulty you experience in speaking due to dryness
0______________________________________________________50 mm
Not difficult at all Very difficult
2. Rate the difficulty you experience in swallowing due to dryness
0______________________________________________________50 mm
Not difficult at all Very difficult
3. Rate how much saliva is in your mouth
0______________________________________________________50 mm
A lot None
4. Rate the dryness of your mouth
0______________________________________________________50 mm
Not dry at all Very dry
5. Rate the dryness of your throat
0______________________________________________________50 mm
Not dry at all Very dry
6. Rate the dryness of your lips
0______________________________________________________50 mm
Not dry at all Very dry
7. Rate the dryness of your tongue
0______________________________________________________50 mm
Not dry at all Very dry
8. Rate the level of your thirst
0______________________________________________________50 mm
Not thirsty at all Very thirsty

The GOHAI, VAS (xerostomia), and STAI questionnaires were applied by a trained researcher.

### Statistical Analysis

Statistical analysis was performed by using SPSS version 24.0 statistical software (Statistical Package for the Social Sciences, IBM). For the results of STAI-State, STAI-Trait, and GOHAI, the Wilcoxon test was applied to compare the time points. For the results of the VAS xerostomia questionnaire, two-way repeated measures ANOVA (analysis of variance) was applied, followed by the Tukey test. The *p* -values lower than 0.05 were considered statistically significant.

## Results

### Patient Demographics

This study included more women (62.5%) than men (37.5%), and their mean age was 65.25 years (range = 60–82 years). Of these participants, 35% of women and 15% of men were in the CD group (mean age = 66.75 years), while 27.5% women and 22.5% men were in the RPD group (mean age = 63.75 years).

### Evaluation of Oral Health-Related Quality of Life

For CD wearers, there was a significant reduction in the scores for questions 1, 2, 4, 5, 6, 9, 10, 11, and 12, and a significant increase in the scores for questions 3 and 7 after 3 months of their rehabilitation ( *p* < 0.05; Table 1 ).

**Table 1:** Mean score ± standard deviation for each question of the General Oral Health Assessment Index questionnaire

	Complete denture			Removable partial denture		
Questions (in the past 3 mo)	Initial	3 mo after insertion	*p* -Value a	Initial	3 mo after insertion	*p* -Value a
How often did you limit the kinds or amounts of food you eat because of problems with your teeth or dentures?	1.62 ± 0.77	1.35 ± 0.59	0.005 b	1.60 ± 0.75	1.30 ± 0.57	0.109
How often did you have trouble biting or chewing any kinds of food such as firm meat or apples?	1.62 ± 0.67	1.35 ± 0.59	0.002 b	2.00 ± 0.86	1.30 ± 0.47	0.006 b
How often were you able to swallow comfortably?	1.25 ± 0.44	1.42 ± 0.59	0.020 b	1.10 ± 0.31	1.65 ± 0.81	0.015 b
How often have your teeth or dentures prevented you from speaking the way you wanted?	1.47 ± 0.68	1.15 ± 0.37	0.002 b	1.70 ± 0.86	1.35 ± 0.67	0.083
How often were you able to eat anything feeling discomfort?	1.62 ± 0.74	1.30 ± 0.57	0.002 b	1.80 ± 0.77	1.30 ± 0.47	0.004 b
How often did you limit contacts with people because of the condition of your teeth or dentures?	1.42 ± 0.71	1.10 ± 0.45	0.004 b	1.50 ± 0.69	1.05 ± 0.22	0.014 b
How often were you pleased or happy with the looks of your teeth and gums, or dentures?	1.92 ± 0.83	2.35 ± 0.67	0.004 b	1.95 ± 0.69	1.45 ± 0.60	0.048 b
How often did you use medication to relieve pain or discomfort from around your mouth?	1.30 ± 0.56	1.15 ± 0.37	0.084	1.35 ± 0.59	1.10 ± 0.31	0.132
How often were you worried or concerned about the problems with your teeth, gums, or dentures?	1.62 ± 0.70	1.25 ± 0.44	0.001 b	1.55 ± 0.69	1.25 ± 0.55	0.130
How often did you feel nervous or self-conscious because of problems with your teeth, gums, or dentures?	1.57 ± 0.67	1.25 ± 0.55	0.001 b	1.45 ± 0.76	1.15 ± 0.37	0.063
How often did you feel uncomfortable eating in front of people because of problems with your teeth or dentures?	1.50 ± 0.67	1.10 ± 0.31	0.001 b	1.50 ± 0.69	1.25 ± 0.55	0.132
How often were your teeth or gums sensitive to hot, cold, or sweets?	1.50 ± 0.64	1.25 ± 0.44	0.026 b	1.75 ± 0.79	1.80 ± 0.62	0.782

Abbreviation: mo (months).

a Wilcoxon's test.

b Significant; *p* <0.0.5.

For RPD wearers, there was a significant reduction in the scores for questions 2, 5, 6 and 7, and a significant increase in the score for question 3 after 3 months of their rehabilitation ( *p* < 0.05; Table 1 ).

### Assessment of State Anxiety

For CD wearers, there was a significant reduction in the scores for questions 3, 6, 7, 12, and 13 ( *p* < 0.05), and a significant increase in the scores for questions 1, 2, 5, 8, 10, 11, 15, 16, 19, and 20 after 3 months of their rehabilitation ( *p* < 0.05; Table 2 ).

**Table 2:** Mean score ± standard deviation for each statement of the State Anxiety Inventory-State Questionnaire

	Complete denture			Removable partial denture		
Statements	Initial	3 mo after insertion	*p* -Value a	Initial	3 mo after insertion	*p* -Value a
I feel calm	2.15 ± 0.99	3.10 ± 0.64	0.001 b	2.60 ± 0.68	2.85 ± 0.99	0.194
I feel secure	2.15 ± 0.81	2.85 ± 0.99	0.001 b	2.45 ± 0.94	2.55 ± 1.05	0.414
I am tense	1.65 ± 1.04	1.25 ± 0.44	0.023 b	1.80 ± 0.62	1.65 ± 0.81	0.317
I am regretful	1.30 ± 0.47	1.20 ± 0.41	0.317	1.60 ± 0.60	1.35 ± 0.74	0.096
I feel at ease	1.90 ± 0.72	2.95 ± 0.60	<0.001 b	2.50 ± 0.76	2.65 ± 0.99	0.257
I feel upset	1.85 ± 0.81	1.25 ± 0.44	0.003 b	1.45 ± 0.60	1.40 ± 0.68	0.564
I am presently worrying over possible misfortunes	1.85 ± 0.67	1.45 ± 0.60	0.046 b	1.70 ± 0.98	1.45 ± 0.76	0.025 b
I feel rested	1.80 ± 0.52	2.95 ± 0.76	<0.001 b	2.45 ± 0.76	2.55 ± 0.89	0.527
I feel anxious	1.80 ± 0.77	1.60 ± 0.60	0.356	1.85 ± 0.81	1.70 ± 0.86	0.317
I feel comfortable	2.05 ± 0.76	3.05 ± 0.60	0.001 b	2.25 ± 0.72	2.55 ± 0.83	0.084
I feel self-confident	2.00 ± 0.72	2.90 ± 0.55	0.001 b	2.45 ± 0.60	2.65 ± 0.87	0.234
I feel nervous	1.90 ± 0.64	1.20 ± 0.41	0.001 b	1.90 ± 0.85	1.65 ± 0.74	0.025 b
I am jittery	1.60 ± 0.75	1.30 ± 0.57	0.034 b	1.70 ± 0.86	1.55 ± 0.89	0.180
I feel “high-strung”	1.45 ± 0.76	1.50 ± 0.76	0.317	1.65 ± 0.99	1.45 ± 0.94	0.046 b
I am relaxed	1.95 ± 0.69	2.75 ± 0.79	0.001 b	2.10 ± 0.64	2.65 ± 0.99	0.013 b
I feel content	2.15 ± 0.49	3.05 ± 0.39	<0.001 b	2.15 ± 0.58	2.55 ± 0.60	0.005 b
I am worried	1.80 ± 0.77	1.55 ± 0.60	0.197	1.95 ± 0.76	1.65 ± 0.99	0.034 b
I feel overexcited and “rattled”	1.45 ± 0.60	1.15 ± 0.37	0.083	1.55 ± 0.76	1.40 ± 0.75	0.180
I feel joyful	2.00 ± 0.72	3.20 ± 0.61	<0.001 b	2.50 ± 0.76	2.85 ± 0.93	0.134
I feel pleasant	2.10 ± 0.72	3.10 ± 0.64	0.001 b	2.40 ± 0.68	2.80 ± 0.83	0.046 b

Abbreviation: mo, months.

a Wilcoxon's test.

b Significant; *p* <0.05.

For RPD wearers, there was a significant reduction in the scores for questions 7, 12, 14, and 17 ( *p* < 0.05), and a significant increase in the scores for questions 15, 16, and 20 after 3 months of their rehabilitation ( *p* < 0.05; Table 2 ).

### Assessment of Trait Anxiety

For CD wearers, there was a significant reduction in the scores for questions 17 and 20 ( *p* < 0.05), and a significant increase in the scores for questions 1, 6, 13, and 16 after 3 months of their rehabilitation ( *p* < 0.05; Table 3 ).

**Table 3:** Mean score ± standard deviation for each statement of the State Anxiety Inventory-Trait questionnaire

	Complete denture			Removable partial denture		
Statements	Initial	3 mo after insertion	*p* -Value a	Initial	3 mo after insertion	*p* -Value a
I feel pleasant	2.40 ± 0.75	2.95 ± 0.51	0.008 b	2.85 ± 0.87	3.35 ± 0.87	0.012 b
I tire quickly	1.90 ± 0.91	1.60 ± 0.68	0.083	2.00 ± 0.92	1.45 ± 0.60	0.012 b
I feel like crying	1.10 ± 0.31	1.10 ± 0.31	1.000	1.60 ± 0.75	1.25 ± 0.44	0.020 b
I wish I could be as happy as others seem to be	1.55 ± 0.60	1.45 ± 0.51	0.317	1.40 ± 0.75	1.30 ± 0.66	0.157
I am losing out on things because I can't make up my mind soon enough	1.65 ± 0.67	1.30 ± 0.47	0.053	1.60 ± 0.94	1.55 ± 0.69	0.705
I feel rested	1.80 ± 0.52	2.90 ± 0.72	<0.001 b	2.30 ± 0.80	3.25 ± 0.79	0.001 b
I am “calm, cool, and collected”	2.60 ± 0.75	2.65 ± 0.59	0.763	2.85 ± 1.09	3.30 ± 0.80	0.029 b
I feel that difficulties are piling up so that I cannot overcome them	1.70 ± 0.66	1.50 ± 0.51	0.157	1.40 ± 0.75	1.25 ± 0.44	0.257
I worry too much over something that really doesn't matter	1.95 ± 0.69	1.60 ± 0.60	0.088	1.55 ± 0.89	1.40 ± 0.68	0.257
I am happy	2.90 ± 0.79	3.10 ± 0.79	0.377	2.95 ± 1.00	3.35 ± 0.67	0.046 b
I am inclined to take things hard	1.75 ± 0.64	1.65 ± 0.59	0.480	1.55 ± 0.89	1.40 ± 0.60	0.257
I lack self-confidence	1.70 ± 0.73	1.35 ± 0.49	0.071	1.45 ± 0.89	1.45 ± 0.60	1.000
I feel secure	2.35 ± 1.09	3.10 ± 0.79	0.005 b	2.55 ± 1.00	3.25 ± 0.79	0.002 b
I try to avoid facing a crisis or difficulty	1.90 ± 0.64	2.00 ± 0.46	0.564	1.85 ± 1.04	1.40 ± 0.68	0.007 b
I feel blue	1.50 ± 0.61	1.35 ± 0.49	0.257	1.45 ± 1.00	1.50 ± 0.69	0.705
I am content	2.45 ± 0.76	2.80 ± 0.77	0.008 b	2.95 ± 1.10	3.45 ± 0.69	0.013 b
Some unimportant thought runs through my mind and bothers me	1.90 ± 0.72	1.50 ± 0.76	0.021 b	1.60 ± 0.99	1.35 ± 0.67	0.059
I take disappointments so keenly that I can't put them out of my mind	1.65 ± 0.67	1.40 ± 0.68	0.059	1.40 ± 0.88	1.45 ± 0.69	0.655
I am a steady person	2.75 ± 0.72	3.00 ± 0.56	0.096	3.05 ± 1.15	3.50 ± 0.76	0.020 b
I get in a state of tension or turmoil as I think over my recent concerns and interests	1.95 ± 0.83	1.60 ± 0.82	0.020 b	1.45 ± 0.89	1.50 ± 0.89	0.655

Abbreviation: mo, months.

a Wilcoxon's test.

b Significant; *p* <0.05.

For RPD wearers, there was a significant reduction in the scores for questions 2, 3, and 14, and a significant increase in the scores for questions 1, 6, 7, 10, 13, 16, and 19 after 3 months of their rehabilitation ( *p* < 0.05; Table 3 ).

### Evaluation of Dry Mouth Sensation

For both groups, all questions of the VAS xerostomia questionnaire showed a significant perception of increased salivation after 3 months of insertion of new dentures ( *p* < 0.05; Table 4 ).

**Table 4:** Mean results (centimeters) for each item of the visual analog scale xerostomia questionnaire

	Complete denture		Removable partial denture	
Items	Initial	3 mo after insertion	Initial	3 mo after insertion
Rate the difficulty you experience in speaking due to dryness	3.72 (1.04) Aa	2.34 (0.80) Ab	1.60 (0.75) Ba	1.30 (0.57) Ab
Rate the difficulty you have in swallowing due to dryness	3.68 (1.13) Aa	2.44 (0.67) Ab	2.00 (0.86) Ba	1.30 (0.47) Ab
Rate how much saliva is in your mouth	3.77 (1.22) Aa	2.45 (0.61) Ab	1.65 (0.81) Ba	1.10 (0.31) Ab
Rate the dryness of your mouth	3.40 (0.98) Aa	2.41 (0.78) Ab	2.41 (0.74) Ba	1.90 (0.66) Ab
Rate the dryness of your throat	3.67 (1.00) Aa	2.61 (1.11) Ab	2.59 (0.65) Ba	2.07 (0.66) Ab
Rate the dryness of your lips	3.71 (0.98) Aa	2.64 (1.05) Ab	2.78 (0.80) Ba	2.16 (0.71) Ab
Rate the dryness of your tongue	3.57 (0.91) Aa	2.70 (1.16) Ab	2.66 (0.73) Ba	2.10 (0.72) Ab
Rate the level of your thirst	3.46 (0.81) Aa	2.68 (0.90) Ab	3.04 (0.92) Ba	2.68 (0.90) Ab

Note: Visual analog scale xerostomia questionnaire. The values in parentheses represent the standard deviation. Group (complete denture and removable partial denture) × time point (initial and 3 months after insertion; *p* < 0.05). Different capital letters in the horizontal indicate a statistically significant difference between groups (complete denture and removable partial denture) for each time point separately; Tukey's test ( *p* < 0.05). Different lowercase letters in the horizontal indicate a statistically significant difference between the time points (initial and 3 months after insertion of dentures) for each group separately; Tukey's test ( *p* <0.05). Abbreviation: mo (months).

## Discussion

Demographic data showed that this study included more women than men in both groups. This result corroborates studies in the literature that also assessed wearers of CDs and RPDs, and found that most of these wearers of removable dentures were females. 5 6 27

According to De Carvalho et al, the GOHAI shows a high level of internal consistency and reliability. 6 For CD wearers, there was a significant reduction in the scores for questions 1, 2, 4, 5, 6, 9, 10, 11, and 12, and a significant increase in the scores for questions 3 and 7 after 3 months of their rehabilitation ( Table 1 ). This showed a significant increase in oral health-related quality of life for CD wearers after this period as follows: (1) greater ease during their chewing; (2) more comfort during their swallowing; (3) greater comfort when they ate food; (4) greater satisfaction with the appearance of their dentures; (5) greater security when they spoke and ate in front of people; (6) greater social contact between them and people; (7) less limitation on the type or amount of food they could eat; (8) less worry and nervousness about their gums and dentures; and (9) less sensitivity of their gums to heat, cold, and sweets.

For RPD wearers, questions 2, 3, 5, 6, and 7 showed a statistically significant difference between the two time points ( Table 1 ). Therefore, 3 months after oral rehabilitation of these RPD wearers, it was possible to observe the following situations (questions 2, 3, 5, and 6): (1) greater ease during their chewing; (2) more comfort during their swallowing; (3) greater comfort when they ate food; and (4) greater social contact between them and people. It is noteworthy that the score for question 7 was negatively influenced by the expectations of these patients. They expected a better aesthetic of their smile, without exposing the metallic parts of their RPDs. However, the exposure of metallic parts of RPDs during the smile is common in oral rehabilitations with this type of denture.

In Table 1 , after 3 months of oral rehabilitation, it is possible to observe that the improvement in quality of life was greater for CD wearers than for RPD wearers. Possibly this occurred due to the following situations:

- Presence of natural teeth in the RPD group. Possibly, RPD wearers' natural teeth helped to preserve much of the retention and stability over time for their old RPDs. On the other hand, due to the continuous resorption process of the bone ridges of CD wearers, 7 their old CDs were very unstable on their arches. Thus, at the end time point, the positive effect of rehabilitation with new dentures (greater retention and stability) was smaller for RPD wearers than for CD wearers, and consequently, this influenced the GOHAI results ( Table 1 ).
- Periodontal ligament receptors play an important role related to jaw movements, chewing food, and controlling the intensity of bite force. 28 Thus, it is suggested that partially edentulous patients have better control of their jaw than completely edentulous patients. 28 Therefore, possibly, RPD wearers had a better functional relationship with their old dentures compared with CD wearers; and this may also have contributed to the fact that RPD wearers had a lower perception of improvement in their quality of life compared with the CD group at the end time point.
- After the insertion of new removable dentures (CDs/RPDs), the aesthetic appearance of the smile generated by the new RPDs was not as visually impressive as the aesthetic appearance of the smile generated by the new CDs. As previously reported, the aesthetic factor had a negative influence on the quality of life of RPD wearers at the end time point.

State anxiety is related to how the individual feels “right now” or “at this moment.” 25 Based on the STAI-State for CD wearers, it is possible to observe that there was a statistically significant difference between the evaluated time points, for questions 1, 2, 3, 5, 6, 7, 8, 10, 11, 12, 13, 15, 16, 19, and 20 ( Table 2 ). Therefore, all these questions showed a significant reduction in the state anxiety of CD wearers after 3 months of their oral rehabilitation, that is, they were (1) calmer, (2) safer, (3) feeling more at ease, (4) feeling more rested, (5) feeling more comfortable, (6) feeling more self-confident, (7) more relaxed, (8) feeling more content, (9) feeling more joyful, (10) feeling more pleasant, (11) less tense, (12) feeling less upset, (13) less worry because of possible misfortunes, (14) feeling less nervous, and (15) less jittery ( Table 2 ). In addition, for RPD wearers ( Table 2 ), there was also a reduction in their state anxiety, that is, they were (1) less worry because of possible misfortunes, (2) feeling less nervous, (3) feeling less “high-strung,” (4) more relaxed, (5) feeling more content, (6) less worry, and (7) feeling more pleasant. It is worth mentioning that the state anxiety level 25 was lower for CD wearers than for RPD wearers after 3 months of their rehabilitation. Possibly as explained above, as the positive effect of oral rehabilitation was smaller for RPD wearers than for CD wearers, and this may have influenced the psychological factor of these patients. Therefore, at the final time point, CD wearers showed a more positive psychological state due to their oral rehabilitation than RPD wearers.

Trait anxiety is related to how the individual generally feels. 25 Table 3 shows that there was a statistically significant difference between the evaluated time points, for questions 1, 6, 13, 16, 17, and 20. Thus, there was a reduction in trait anxiety for CD wearers at the end time point, that is, they were (1) feeling more pleasant, (2) feeling more rested, (3) feeling more secure, (4) more content, (5) less negatively influenced by unimportant thoughts, and (6) less tense when thinking about their concerns. In addition, there was a reduction in trait anxiety for RPD wearers at the end time point (questions 1, 2, 3, 6, 7, 10, 13, 14, 16, and 19), that is, these individuals were (1) feeling more pleasant; (2) feeling more rested; (3) calmer, nicer, and more controlled; (4) happier, (5) feeling safer, (6) more content, (7) more steady, (8) tiring more slowly, (9) feeling less like crying; and (10) facing more of their crises or difficulties.

Based on the last paragraphs, after 3 months of insertion of new dentures, CD wearers showed a greater perception of improvement in their quality of life and psychological state (STAI-State) than RPD wearers. Thus, the dentist should be aware of these differences between these two types of patients, paying special attention to RPD wearers and explaining to them the improvements obtained after their oral rehabilitation.

In Table 4 , initially, there was a difference for all situations between the CD and RPD groups. Thus, RPD wearers had significantly lower dry mouth perception than CD wearers. One possible explanation is that these RPD wearers used a toothbrush to clean their natural teeth every day after their meals, and this stimulated their salivation. 29 30 On the other hand, possibly, the evaluated CD wearers did not frequently use a toothbrush to clean their mouth due to their complete edentulism, and consequently their salivation was less stimulated compared with RPD wearers. Therefore, during rehabilitation of CD wearers, it is important to encourage these patients to brush their oral tissue with a toothbrush to stimulate their salivation.

In this study, there was a significant perception of greater salivation in both groups after 3 months of insertion of new dentures ( Table 4 ). It is speculated that new removable dentures act as foreign bodies in the mouth, stimulating salivation. 31 Therefore, this information can explain this result in Table 4 .

## Conclusion

For both groups, it was observed that 3 months after the insertion of new removable dentures:

- There was an increase in oral health-related quality of life.
- There was a reduction in anxiety (trait anxiety and state anxiety).
- There was a perception of greater salivation.
